# Drug and xenobiotic biotransformation in the blood–brain barrier: a neglected issue

**DOI:** 10.3389/fncel.2014.00335

**Published:** 2014-10-17

**Authors:** José A. G. Agúndez, Félix J. Jiménez-Jiménez, Hortensia Alonso-Navarro, Elena García-Martín

**Affiliations:** ^1^Department of Pharmacology, University of ExtremaduraCáceres, Spain; ^2^ISCIII Research Network of Adverse Reactions to Allergens and DrugsMadrid, Spain; ^3^Section of Neurology, Hospital Universitario del SuresteMadrid, Spain; ^4^Department of Biochemistry, Molecular Biology and Genetics, University of ExtremaduraCáceres, Spain

**Keywords:** blood–brain barrier, drug metabolizing enzymes, expression profiling, CNS drug, drug response

## Abstract

Drug biotransformation is a crucial mechanism for facilitating the elimination of chemicals from the organism and for decreasing their pharmacological activity. Published evidence suggests that brain drug metabolism may play a role in the development of adverse drug reactions and in the clinical response to drugs and xenobiotics. The blood–brain barrier (BBB) has been regarded mainly as a physical barrier for drugs and xenobiotics, and little attention has been paid to the BBB as a drug-metabolizing barrier. The presence of drug-metabolizing enzymes in the BBB is likely to have functional implications because local metabolism may inactivate drugs or may modify the drug’s ability to cross the BBB, thus modifying drug response and the risk of developing adverse drug reactions. In this perspective paper, we discuss the expression of relevant xenobiotic metabolizing enzymes in the brain and in the BBB, and we cover current advances and future directions on the potential role of these BBB drug-metabolizing enzymes as modifiers of drug response.

The concept of the blood–brain barrier (BBB) was developed over a century ago, when Ehrlich observed that dyes administered intravenously do not stain the brain. Goldmann refuted the so-called “binding hypothesis” (Goldmann, 1913) and established that the central nervous system (CNS) is separated from the bloodstream by the blood–brain and cerebro-spinal fluid (CSF) barriers. At present, most of the research related to the BBB has focused on how selected molecules, drugs, metabolites, and toxic substances are able to enter and leave the brain (for a recent review, see Pardridge, 2012). In fact, the BBB has been traditionally regarded as a physical barrier that protects the CNS from non-lipophilic drugs and xenobiotics. In contrast, the putative role of the BBB as a drug-metabolizing barrier has received little attention. The presence of drug-inactivating enzymes in the BBB is likely to affect drug response, as does the presence of such enzymes in the intestine and liver (Pereira de Sousa and Bernkop-Schnurch, 2014). Local metabolism may modify the response and the risk of developing adverse drug reactions with drugs affecting the CNS, and the presence of drug-inactivating enzymes in the BBB may constitute an additional protecting mechanism against drugs and xenobiotics, which may act regardless of their lipophilicity.

With the obvious quantitative differences in enzyme expression of drug-inactivating enzymes between the BBB and the functional unit responsible for pre-systemic metabolism [intestinal enzymes (Pereira de Sousa and Bernkop-Schnurch, 2014), gut microbiota (Kang et al., 2013), and first pass in the liver], drug inactivation in the BBB may constitute a relevant quantitative or qualitative factor for CNS drugs, if these are metabolized by enzymes expressed in the BBB. In fact, a relevant problem in the study of drug response, with regard to drugs affecting the CNS, is related to large interspecies differences in drug bioavailability and distribution within the CNS, including differences between primates and humans; these differences, which would not be expected in a purely physical barrier, are likely to be related to differences in the BBB function. Regarding CNS drugs, this variability could be the consequence of variation in the expression patterns and function of drug-metabolizing enzymes and transporters in the BBB. Of great interest is the development of *in vitro* BBB models using brain vascular endothelial cell cultures which permit the characterization and quantification of genes and proteins in brain microvessels from different species (Shawahna et al., 2013). This methodological advance, together with *in vivo* studies comparing drug concentration and metabolic profiles on both sides of the BBB, will contribute in coming years to greater knowledge of the metabolic and functional implications of the BBB.

## ENZYMES THAT METABOLIZE CNS DRUGS

The BBB expresses a variety of neurotransmitter-metabolizing enzymes such as monoamine oxidases (MAO), catechol *O*-methyl transferase (COMT), cholinesterases, GABA transaminase, aminopeptidases, and endopeptidases. Several drug- and xenobiotic-detoxicating enzymes are found in brain capillaries (Minn et al., 1991; de Leon, 2003; Granberg et al., 2003; Haseloff et al., 2006; Ueno, 2009; Wang et al., 2011), thus constituting an enzymatic mechanism that protects the brain from circulating neurotransmitters and from drugs and toxins. For many CNS drugs, metabolic predispositions are a crucial mechanism in determining drug effects. Among the enzymes involved in drug metabolism, two main enzyme categories (Phase I and Phase II) exist. The most relevant Phase I enzymes, in terms of percentage of drugs that they metabolize, are the cytochrome P450 (CYP) CYP3A (including CYP3A4 and CYP3A5, which share several substrates), CYP2D6, CYP2C9 [which share several substrates with CYP2C8, particularly non-steroidal anti-inflammatory drugs (Agundez et al., 2009)], CYP1A1/1A2 and, to a lesser extent, CYP2C19, CYP2E1, CYP1B1, and other CYP enzymes (Evans and Relling, 1999). Regarding Phase II enzymes, the most relevant in terms of percentage of drugs metabolized are UDP-glucuronyltransferases (UDPs), glutathione *S*-transferases (GSTs), sulphotransferases (SULTs), and *N*-acetyltransferases (NATs) particularly NAT2 (Evans and Relling, 1999). Drugs belonging to many pharmacological groups are affected by polymorphic metabolism mediated by these enzymes, including for instance anesthetics, anti-Parkinson’s disease drugs, antihistamine drugs, antipsychotics, narcotics, or antidepressants; specific recommendations for the use of these drugs in the context of variability in drug metabolism have been published (Restrepo et al., 2009; Khokhar and Tyndale, 2011; Relling and Klein, 2011; Swen et al., 2011; Agundez et al., 2013; Garcia-Martin et al., 2013).

## DRUG-METABOLIZING ENZYMES IN THE BRAIN

Most available information on the expression of drug-metabolizing enzymes in the CNS corresponds to the whole brain. The expression of CYP450 in the whole brain was reported by Nishimura et al. (2003). Dutheil et al. (2009) showed the apparent selective expression in several cerebral regions in both neuronal and glial cells. The most relevant enzymes were CYP1B1, CYP2D6, CYP2E1, CYP2J2, CYP2U1, and CYP46A1, with heterogeneous distribution in different brain areas (Dutheil et al., 2009). Tissue-specific features are becoming apparent from recent studies. For instance, the dura-mater is clearly different from the other brain structures because of its particular pattern expression of CYP450, with a high level of CYP1B1 and, to a lesser extent CYP1A1, CYP2U1, CYP3A5, CYP2R1, CYP2E1, CYP2D6, and CYP46A1 (Dutheil et al., 2009). The European Bioinformatics Institute Expression Atlas^1^ (available at the website), provides interesting information on the baseline expression of drug-metabolizing enzymes in the human brain. When comparing the expression in the brain and in the liver, what is striking is the high relative expression in the brain of the Phase I enzymes CYP46A1 (which is virtually absent in the liver and highly expressed in the brain), CYP1B1 and CYP2U1, which are expressed at twice the level in the brain as in the liver. CYP2R1 is expressed in a similar extent in the brain and the liver, and CYP2J2 is expressed in the brain at about 10% of the level in the liver. CYP2D6 has a marginal expression in the brain, representing about 2% of the liver levels, whereas the expression of other CYPs such as CYP1A1, CYP2C8, CYP2C9, CYP2C19, CYP3A4, CYP3A5, and CYP2E1 seems to be negligible in the human brain in basal conditions. Regarding Phase II enzymes, the expression atlas indicates that a high expression of GST enzymes is present in the human brain; specifically, a 10-fold higher expression of GSTM2, an eightfold higher expression of GSTM3, a fourfold higher expression of GSTP1 and GST4, a twofold higher expression of COMT, and about half of the expression levels of SULT1A4, as compared with the liver, respectively. No significant expression of other relevant drug-metabolizing enzymes, such as SULT1A1, UGT1A6, UGT2B7, NAT1, and NAT2 seems to occur in the human brain. Nevertheless, many of the mentioned drug-metabolizing enzymes are inducible and hence, basal values should be considered as reference values, but cannot be extrapolated to all individuals and to all situations.

## DRUG-METABOLIZING ENZYMES IN THE BBB

Dauchy et al. (2008) identified CYPs in microvessels and emphasized the quantitative importance of CYP1B1 in the BBB. Shawahna et al. (2011) quantified the expression of the genes encoding Phase I and Phase II metabolizing enzymes and proteins in brain microvessels from 12 patients suffering from epilepsy or glioma. CYP1B1 and CYP2U1 transcripts were the main CYPs detected in brain microvessels whereas no other CYP proteins were detected (Decleves et al., 2011; Shawahna et al., 2011). Drug-metabolizing enzymes present in microvessels (at a protein detection level) were CYP1B1, CYP2U1, GSTP1, GSTM2, GSTM3, GSTM5, and GSTO1. In addition, detectable MRNA corresponding to CYP2D6, CYP2J2, CYP2E1, CYP2R1 were present, as well as the Phase II enzymes histamine *N*-methyltransferase (HNMT), COMT, and thiopurine *S*-methyltransferase (TPMT; Shawahna et al., 2013). Conversely, no UGTs, or NAT enzymes were identified in microvessels (Shawahna et al., 2013), which is consistent with the virtual absence of expression in the human brain according to The European Bioinformatics Institute Expression Atlas^1^. According to present knowledge, the metabolic capacity of the BBB is likely to modify drug response and, in particular, may be involved in therapeutic failure for drugs that are substrates of the enzymes present in the BBB. Not only because the drugs may be chemically inactivated, but also because drug metabolism at the BBB may modify drug polarity, making the molecules unable to cross the BBB. Because glial cells form a physical barrier in the BBB, the presence of drug-metabolizing enzymes in astrocytes and microglia constitute a line of defense that drugs cannot avoid when entering the CNS. An exhaustive list of drugs and substrates of drug-metabolizing enzymes present in the BBB falls beyond the scope of a perspective article, but some examples are the following: CYP1B1 metabolizes, among other substrates, amodiaquine, caffeine, theophylline, melatonin, and procarbazine (Shimada et al., 1997, 1999; Li et al., 2000, 2002; Spink et al., 2000; Bournique and Lemarie, 2002; Patterson and Murray, 2002; Choudhary et al., 2004; Dubey et al., 2005; Ma et al., 2005; Zhang et al., 2007), CYP46A1 participates in the metabolism of analgesics such as dextromethorphan, diclofenac, or phenacetin (Mast et al., 2003); CYP2D6 participates in the metabolism of several antidepressive agents, antipsychotics, and other CNS drugs (for a recent review, see Agúndez and García-Martin, 2014). CYP2J2 metabolizes ergocalciferol, ebastine, and astemizole (Hashizume et al., 2002; Matsumoto et al., 2002, 2003; Lee et al., 2005; Zhou et al., 2005; Aiba et al., 2006). CYP2E1 participates in the metabolism of anesthetics and ethanol (Zakhari, 2006; Restrepo et al., 2009; Martinez et al., 2010). CYP2R1 participates in the metabolism of ergocalciferol and colecalciferol (Schuster, 2011). HNMT metabolizes histamine, particularly in the CNS, and *HNMT* gene variations are relatively common and affect the enzyme activity (Garcia-Martin et al., 2009). GST enzymes in the BBB impair accumulation and cause therapeutic failure for antiepileptic drugs (Shang et al., 2008).

## CLINICAL IMPLICATIONS AND FUTURE PERSPECTIVES

In contrast to the extensive investigation of drug-metabolizing enzymes in the human liver carried out in the last three decades, and compared to the present knowledge of drug transporters in the BBB, the implications of drug-metabolizing enzymes in the BBB are poorly understood. These enzymes may be a major cause of dissociation between the drug concentrations observed in the CSF and plasma, and may underlie therapeutic failure, even when plasma drug concentrations are optimal. Several issues that require further investigation include the following:

(1) Identification and quantification of all drug-metabolizing enzymes in the BBB. So far our knowledge is very limited and further studies are required to identify more enzymes, to analyze their expressions in different structures in the BBB, and to study the interindividual variability in the expression of these enzymes.
(2) Specific characteristics of the drug-metabolizing enzymes expressed in the BBB. The first exhaustive gene profiling of P450 in human brain microvessels was carried out by Dauchy et al. (2008). According to the 1000 genomes catalog^2^ (available at the website), most of these enzymes show several splice variants. For instance, CYP1B1 has seven transcripts, two of which encode full-length protein, CYP2U1 has three transcripts, two of these with protein product, GSTP1 has nine transcripts, GSTM has fourteen, GSTM3 and GSTM5 have six each, and GSTO1 has seven. With the exception of CYP2D6, which has only one known transcript, the enzymes detected in the BBB at mRNA level also have several transcripts: CYP2J2 has five transcripts (although only one functional), CYP2E1 and CYP2R1 have ten transcripts each. It is crucial to know which transcripts are expressed in the BBB, both under basal conditions and in CNS or vascular disorders, as well as their characteristics (substrate specificity, Vmax, or Km).
(3) Mechanisms involved in the regulation and functional effects of drug-metabolizing enzymes in the BBB: Effects of known inducers of liver enzymes on BBB drug-metabolizing enzymes, the effect of gene variations, and factors underlying the inter-individual variability in enzyme activity.
(4) Effects of known inhibitors of the liver enzymes on the BBB enzymes. This is a crucial factor that may underlie drug interactions which cannot be assessed by conventional therapeutic drug monitoring, that is, by determination of drug concentration in plasma.

In summary, besides acting as a physical barrier, the BBB constitutes a highly specialized metabolic barrier, and contains several drug-metabolizing enzymes, many of which have the ability to inactivate drugs and toxins before they enter the CNS. According to the specific pattern of enzymes, the BBB metabolic barrier has a different metabolic profile than that of pre-systemic metabolism, where CYP3A4 and CYP3A5 enzymes play a key role. These CYP3A enzymes, which show little selectivity since they are involved in the metabolism of a high percentage of clinically used drugs (Evans and Relling, 1999), are not present in the BBB (Shawahna et al., 2011). The metabolic BBB barrier seems to be selective for specific types of drugs or xenobiotics that are metabolized by the enzymes present in the BBB. Nevertheless, much additional information is necessary to gain more ground in BBB metabolism and it is expected that in the coming years we will have new information available for assessing the potential and clinical implications of local drug metabolism in the BBB, which so far have received little attention.

## Conflict of Interest Statement

The authors declare that the research was conducted in the absence of any commercial or financial relationships that could be construed as a potential conflict of interest.

## Footnotes

1 http://www.ebi.ac.uk/gxa/home

2 http://browser.1000genomes.org/Homo_sapiens/Search/New?db=core
